# GTAT-GRN: a graph topology-aware attention method with multi-source feature fusion for gene regulatory network inference

**DOI:** 10.3389/fgene.2025.1668773

**Published:** 2025-10-08

**Authors:** Shuran Wang, Lilian Zhang, Lutao Gao, Yao Rao, Jie Cui, Linnan Yang

**Affiliations:** ^1^ College of Big Data, Yunnan Agricultural University, Kunming, China; ^2^ Yunnan Engineering Technology Research Center of Agricultural Big Data, Kunming, China; ^3^ Yunnan Engineering Research Center for Big Data Intelligent Information Processing of Green Agricultural Products, Kunming, China

**Keywords:** gene regulatory network, graph neural network, topology-aware attention mechanism, feature fusion, network inference

## Abstract

Gene regulatory network (GRN) inference is a central task in systems biology. However, due to the noisy nature of gene expression data and the diversity of regulatory structures, accurate GRN inference remains challenging. We hypothesize that integrating multi-source features and leveraging an attention mechanism that explicitly captures graph structure can enhance GRN inference performance. Based on this, we propose GTAT-GRN, a deep graph neural network model with a graph topological attention mechanism that fuses multi-source features. GTAT-GRN includes a feature fusion module to jointly model temporal expression patterns, baseline expression levels, and structural topological attributes, improving node representation. In addition, we introduce the Graph Topology-Aware Attention Network (GTAT), which combines graph structure information with multi-head attention to capture potential gene regulatory dependencies. We conducted comprehensive evaluations of GTAT-GRN on multiple benchmark datasets and compared it with several state-of-the-art inference methods, including GENIE3 and GreyNet. The experimental results show that GTAT-GRN consistently achieves higher inference accuracy and improved robustness across datasets. These findings indicate that integrating graph topological attention with multi-source feature fusion can effectively enhance GRN reconstruction.

## 1 Introduction

Genes are the fundamental carriers of genetic information in cells. By encoding proteins, they drive and regulate a wide range of cellular processes. Gene function extends beyond protein coding. Through complex gene regulatory network (GRN), genes precisely modulate cellular behavior and functional states. A GRN is an intricate system that controls gene expression inside the cell (Kaler et al., 2009). Reconstructing this network is essential to modern biology. By mapping gene-gene interactions, a GRN exposes the dynamic control of gene expression across environmental conditions and developmental stages (Davidson, 2010). GRN reconstruction not only clarifies basic principles of life (Jie et al., 2020) but also underpins studies of disease mechanisms and the discovery of drug targets (Morgan et al., 2020). In cancer research, GRN analysis reveals transcription factors such as p53 (Kurup et al., 2023) and MYC that drive tumorigenesis, along with their downstream networks. These insights inform the design of personalized therapies. In developmental biology, GRN dissection uncovers core modules (Bedois et al., 2021), such as the HOX gene cluster, that govern organ formation and advance regenerative medicine research.

However, conventional GRN inference methods still confront several challenges. Chief among these is their high computational complexity. As genomic datasets grow, traditional algorithms, such as those based on mutual information (Zhang et al., 2015) or regression (Adabor and Acquaah-Mensah, 2019), scale poorly and slow dramatically on large inputs. Data sparsity is another barrier to accurate GRN reconstruction. Because techniques like ChIP-seq validate only a subset of interactions, many gene-gene links remain unconfirmed, yielding incomplete networks (McCalla et al., 2023). Moreover, conventional methods (e.g., Pearson correlation (Butte and Kohane, 1999) and linear regression (Lèbre et al., 2010)) assume linear dependencies, so they miss nonlinear regulatory relationships and further degrade inference accuracy.

In recent years, GNN has demonstrated considerable potential for inferring GRN owing to its strong capacity to learn from graph structures (Paul et al., 2024). GRGNN (Wang et al., 2020) was the first framework to cast GRN inference as a graph-classification problem, thereby introducing GNN to GRN research. Because GNN operates natively on graphs, it is well suited to model the complex regulatory relationships among genes. Moreover, its strong capacity to generalize enables GNN to extract latent regulatory patterns from limited experimental data, conferring greater robustness and scalability on GRN inference (Paul et al., 2024). Nonetheless, current GNN-based approaches typically rely on predefined graph structures or shallow attention mechanisms and therefore fail to capture the full spectrum of latent topological information among genes (Liu et al., 2023).

Inspired by the limitations of existing approaches, this study proposes a novel GRN inference model, termed GTAT-GRN, which is based on the Graph Topology-Aware Attention Network (GTAT) (Shen et al., 2025). Unlike conventional methods that rely on predefined graph structures or shallow attention mechanisms, GTAT-GRN integrates multi-source feature fusion with topology-aware modeling to enhance the ability to capture complex regulatory relationships. Specifically, the model incorporates a multi-feature fusion module that jointly encodes temporal expression patterns, baseline expression levels, and potential topological attributes of genes, thereby achieving heterogeneous feature integration (Yu et al., 2024b) and enriching node representations with multidimensional expressiveness. Meanwhile, the introduced GTAT dynamically captures high-order dependencies and asymmetric topological relationships among genes during graph learning, thereby uncovering latent regulatory patterns more effectively.

Accordingly, the central hypothesis of this work is that by systematically integrating multi-source biological features and employing a topology-aware attention mechanism to explicitly model topological dependencies among genes, it is possible to substantially improve the characterization of true GRN structures and the accuracy of network inference.

The main contributions of this paper are as follows.

1. We propose GTAT-GRN, a graph-topology-attention model that accurately infers gene regulatory networks by learning inter-gene topological relationships.
2. By fusing topological cues with complementary temporal and static features, GTAT-GRN integrates multidimensional information to decode gene regulation.
3. The model was systematically evaluated on the DREAM4 and DREAM5 standard datasets. Experimental results indicate that GTAT-GRN outperforms existing methods across overall metrics, including AUC and AUPR. Moreover, it demonstrates high-confidence predictive performance on Top-k metrics (Precision@k, Recall@k, F1@k), confirming its validity, robustness, and capacity to capture key regulatory relationships across different datasets.

## 2 Materials and methods

**FIGURE 1:**
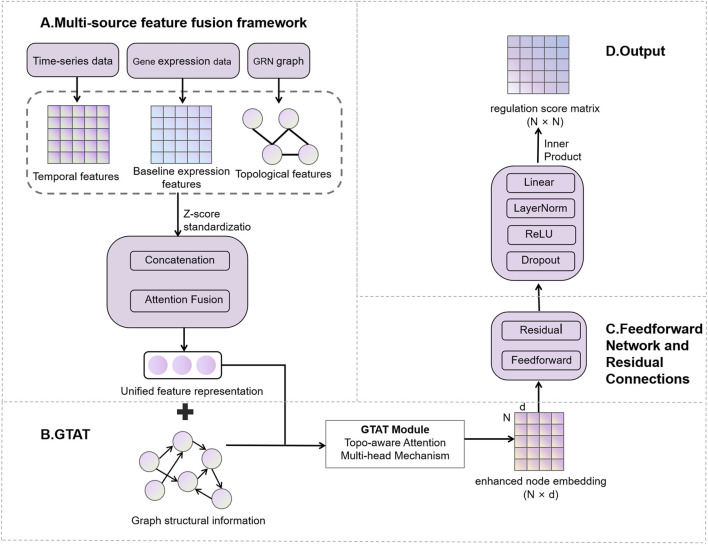
Overview of the GTAT-GRN framework.

The proposed GTAT-GRN method is a novel GRN inference approach based on GTAT (Shen et al., 2025). The architecture of GTAT-GRN is shown in Figure 1, consisting of four modules: (A) multi-source feature fusion framework, (B) Graph Topology Attention Network (GTAT), (C) feedforward network and residual connections, and (D) GRN prediction output layer.

### 2.1 Multi-source feature fusion framework

To improve GRN inference, we design a multi-source feature-fusion module (Wu et al., 2023) that jointly models three information streams: temporal dynamics of gene expression (Rubiolo et al., 2015), baseline expression patterns (Yuan and Bar-Joseph, 2021), and network topology (Liu et al., 2023). The types, sources, treatment methods and biological functions of the features are detailed in Table 1.

**TABLE 1:** Feature types and their related informatio**n**.

Feature type	Data source	Treatment method	Biological significance
Temporal features	Gene expression time series data	Extract statistical indicators (such as mean, standard deviation, maximum value, etc.)	Reflect the dynamic changes in gene expression and reveal the expression levels and changing trends of genes at different time points
Expression-profile features	Gene expression data under wild-type or multiple conditions	Calculate the statistical characteristics such as expression level, stability and specificity	Describe the expression characteristics of genes under different conditions and provide background information for inferring their regulatory roles
Topological features	The structure of the gene regulatory network diagram	Calculate indicators such as degree centrality, in-degree, out-degree, and clustering coefficient	Reveal the structural role of genes in the network and capture the regulatory relationships and interactions among genes

#### 2.1.1 Feature description

##### 2.1.1.1 Temporal features

     Temporal features characterize gene-expression levels at discrete time points and the trajectories of their changes over time (Huynh-Thu and Geurts, 2018). These descriptors capture dynamic expression patterns and furnish critical cues for inferring gene-regulatory relationships. Key metrics extracted are as follows.

• Mean: Summarizes the overall expression level.

• Standard deviation: Quantifies expression variability.

• Maximum and minimum: Define the extreme range of expression.

• Skewness: Measures distributional asymmetry.

• Kurtosis: Measures the peakedness of the expression distribution.

• Time-series trend: Delineates the directional change in expression over time.

##### 2.1.1.2 Expression-profile features

     Expression-profile features summarize gene-expression levels and their variation across basal and diverse experimental conditions (Yuan and Bar-Joseph, 2021). They facilitate analyses of gene-expression stability, context specificity, and potential functional pathways, thereby supplying essential context for inferring regulatory roles. Key metrics derived from baseline-expression features include.

• Baseline expression level: The gene’s expression in wild-type (control) conditions.

• Expression stability: The degree of variation in expression across conditions.

• Expression specificity: The extent to which a gene is preferentially expressed in particular conditions.

• Expression pattern: The qualitative profile of expression changes across multiple conditions.

• Expression correlation: The pairwise correlation of expression levels between genes.

##### 2.1.1.3 Topological features

     Topological features are derived from the structural properties of nodes in a GRN (Gene Regulatory Network) graph; they characterize each gene’s position, importance, and interactions with other genes (Pham et al., 2024). In a GRN, genes are represented as nodes and regulatory relationships as edges. Computing these topological descriptors allows us to elucidate gene functions within the network, trace how regulatory signals propagate, and pinpoint key hub genes. Key metrics include.

• Degree centrality: Counts the total number of direct regulatory links a gene has.

• In-degree: The number of regulators targeting the gene.

• Out-degree: The number of targets regulated by the gene.

• Clustering coefficient: Measures the cohesiveness of the gene’s local neighborhood.

• Betweenness centrality: Quantifies the gene’s hub role by capturing its control over information flow.

• Local efficiency: Evaluates the efficiency of information transfer within the gene’s immediate neighborhood.

• PageRank score: Assigns an importance value based on the gene’s influence in the network.

• k-core index: Indicates the gene’s membership within progressively denser network cores.

Together, these topological measures expose the structural roles of genes in a GRN and facilitate the discovery of regulatory interactions.

#### 2.1.2 Feature extraction and preprocessing

##### 2.1.2.1 Temporal features extraction

     Temporal features are extracted from gene expression time-series data $X_{t}\in R^{N\times T}$ , where N represents the number of genes and T represents the number of time points. For each gene’s time-series expression data, Z-score normalization (Mare et al., 2017) is applied to ensure that each gene has zero mean and unit variance across time points. The normalization is performed as follows:

$${\hat{X}}_{t}\left(i,:\right)=\frac{X_{t}\left(i,:\right)-\mu_{i}}{\sigma_{i}}$$ (1)

where $\mu_{i}$ and $\sigma_{i}$ denote the mean and standard deviation of gene i ’s expression values across all time points, respectively. Equation 1 ensures that each gene’s expression profile is standardized, facilitating fair comparison across genes during model training.

##### 2.1.2.2 Baseline expression feature extraction

     Baseline expression features are extracted from wild-type expression data, typically including mean, standard deviation, and other statistical measures. These features are computed to form an expression feature vector for each gene $X_{b}\in R^{N\times d^{b}}$ , where $d^{b}$ represents the feature dimension.

##### 2.1.2.3 Topological features extraction

     Topological features are extracted from the GRN, reflecting each gene’s structural position and importance within the network. These features include degree centrality, in-degree, out-degree, and other metrics that reveal each gene’s role and influence in the regulatory network. Topological features are $X_{g}\in R^{N\times d^{g}}$ computed from the GRN graph G=(V,E) , where V represents gene nodes and E represents regulatory edges between genes. For each gene, we compute network metrics such as degree centrality, in-degree, and out-degree. These features are normalized using Z-score standardization to ensure consistency in scale with other features.

##### 2.1.2.4 Feature alignment and normalization

     To ensure that the three feature types are within the same numerical range, we apply Z-score standardization to eliminate scale differences across features (Zhu et al., 2021). Additionally, we handle missing values and standardize the sample dimensions to ensure that features can be fused across the same sample set.

#### 2.1.3 Feature fusion

After feature extraction and preprocessing, the feature fusion phase begins.

1. Primary fusion (concatenation): The three types of features are concatenated along the feature dimension to form a unified feature representation (Ko et al., 2025):

$$X_{\text{concat}}=\left[{\hat{X}}_{t}\|{\hat{X}}_{b}\|{\hat{X}}_{g}\right]\in R^{N\times \left(d_{t}+d_{b}+d_{g}\right)}$$ (2)

Here, ${\hat{X}}_{t},{\hat{X}}_{b},{\hat{X}}_{g}$ represent the standardized temporal, baseline expression, and topological features respectively. Equation 2 ensures that all feature modalities are integrated into a single matrix for subsequent processing.

2. Attention mechanism fusion: To learn the importance of each feature modality, an attention mechanism is applied to compute weights $\alpha_{t},\alpha_{b},\alpha_{g}$ (Choi and Lee, 2023):

$$\alpha_{i}=\text{softmax}\left(W_{a}\cdot X_{i}+b_{a}\right),\;i\in \left\{t,b,g\right\}$$ (3)

Equation 3 adaptively learns attention weights for each feature type, allowing the model to focus on the most informative aspects.

3. Weighted feature fusion: Based on the learned attention scores, the final fused feature representation is computed via weighted summation:

$$X_{\text{fused}}=\alpha_{t}⊙{\hat{X}}_{t}+\alpha_{b}⊙{\hat{X}}_{b}+\alpha_{g}⊙{\hat{X}}_{g}$$ (4)

As shown in Equation 4, the fusion process combines all features proportionally according to their attention scores.

4. Feature transformation: The fused features are then passed through a ReLU-activated linear transformation to enhance non-linear representation capability:

$$Z=\text{ReLU}\left(W_{f}\cdot X_{\text{fused}}+b_{f}\right)$$ (5)

Equation 5 enables the network to capture complex relationships between fused features.

5. Gating mechanism: To control information flow, a gating mechanism is introduced to selectively filter the transformed features:

$$Z_{\text{gate}}=Z⊙\sigma \left(W_{g}\cdot X_{\text{fused}}+b_{g}\right)$$ (6)

In Equation 6, the sigmoid function σ(⋅) determines the flow strength of each feature dimension.

6. Dimensionality reduction and residual connection: Finally, the output is computed by applying dimensionality reduction and adding a residual connection to preserve original information (Akhtar et al., 2025):

$$Z_{\text{final}}=\text{Dropout}\left(W_{o}\cdot Z_{\text{gate}}+b_{o}\right)+X_{\text{fused}}$$ (7)

As shown in Equation 7, the residual connection ensures that the original fused features are preserved alongside the transformed output, improving model stability.

### 2.2 Graph Topology Attention Network (GTAT)

The GTAT (Shen et al., 2025) is a variant of GNN that integrates structural information of the graph with attention mechanisms. It is designed to more effectively capture complex dependencies between nodes and enhance both the expressiveness and robustness of graph representation learning. The core idea of GTAT lies in leveraging not only node feature representations but also explicitly incorporating topological attributes of the graph, such as node degree, shortest path length, and adjacency relationships, into a topology-aware attention mechanism (Huang et al., 2022). This integration enhances the model’s capacity to represent and generalize over complex graph structures.

Traditional graph neural networks primarily rely on neighborhood aggregation strategies, performing well in modeling local structures, but they tend to suffer from issues such as over-smoothing, overfitting to noisy edges, and poor robustness to connection patterns when handling higher-order topological relationships and complex regulatory structures (Zhang et al., 2024). To address these issues, GTAT introduces topological priors and combines multi-head attention mechanisms, achieving a better balance between modeling graph structure and integrating feature information (Wang et al., 2025).

1. Topological Feature Modeling: We explicitly extract topological features such as node degree, common neighbors, and shortest path length, which are input as topological features directly involved in attention weight calculation (Zhao et al., 2022). Unlike traditional attention mechanisms that rely solely on node features, this mechanism encodes structural information into the weight allocation process, effectively enhancing the attention mechanism’s sensitivity to structural differences (Xiong et al., 2025). These topological features are consistent with those extracted in the earlier feature fusion module, ensuring the reuse and consistency of structural information across different stages, thereby enhancing the model’s expressive efficiency.
2. Topology-Aware Attention Mechanism: The model incorporates topological features during attention computation to assist in determining the strength of node relationships (Matinyan et al., 2024). Compared to standard Graph Attention Networks (GAT), which may misclassify nodes with similar features but unrelated structures, GTAT, through its topology-aware attention mechanism, demonstrates superior discriminative power and noise resilience in sparsely connected regions.
3. Directed Graph Modeling: EnhancedGCA uses the edge_index parameter to explicitly represent the directed graph structure, a tuple (src,dst) representing directed edges from source to target nodes. During attention computation, the model distinguishes between “regulatory source nodes” and “target nodes”, aligning more closely with the real gene regulatory mechanisms (Naldi et al., 2018). The introduction of multi-head attention enables the model to learn different regulatory patterns in parallel from multiple subspaces, further enhancing its modeling capability (Yu et al., 2024a).
4. Efficient Structure-Aware Aggregation: During attention allocation, by jointly considering node features and their structural context, the model focuses more on structurally similar or biologically meaningful regulatory pathways, effectively mitigating the influence of noisy or irrelevant connections (Cheng et al., 2025).

**FIGURE 2:**
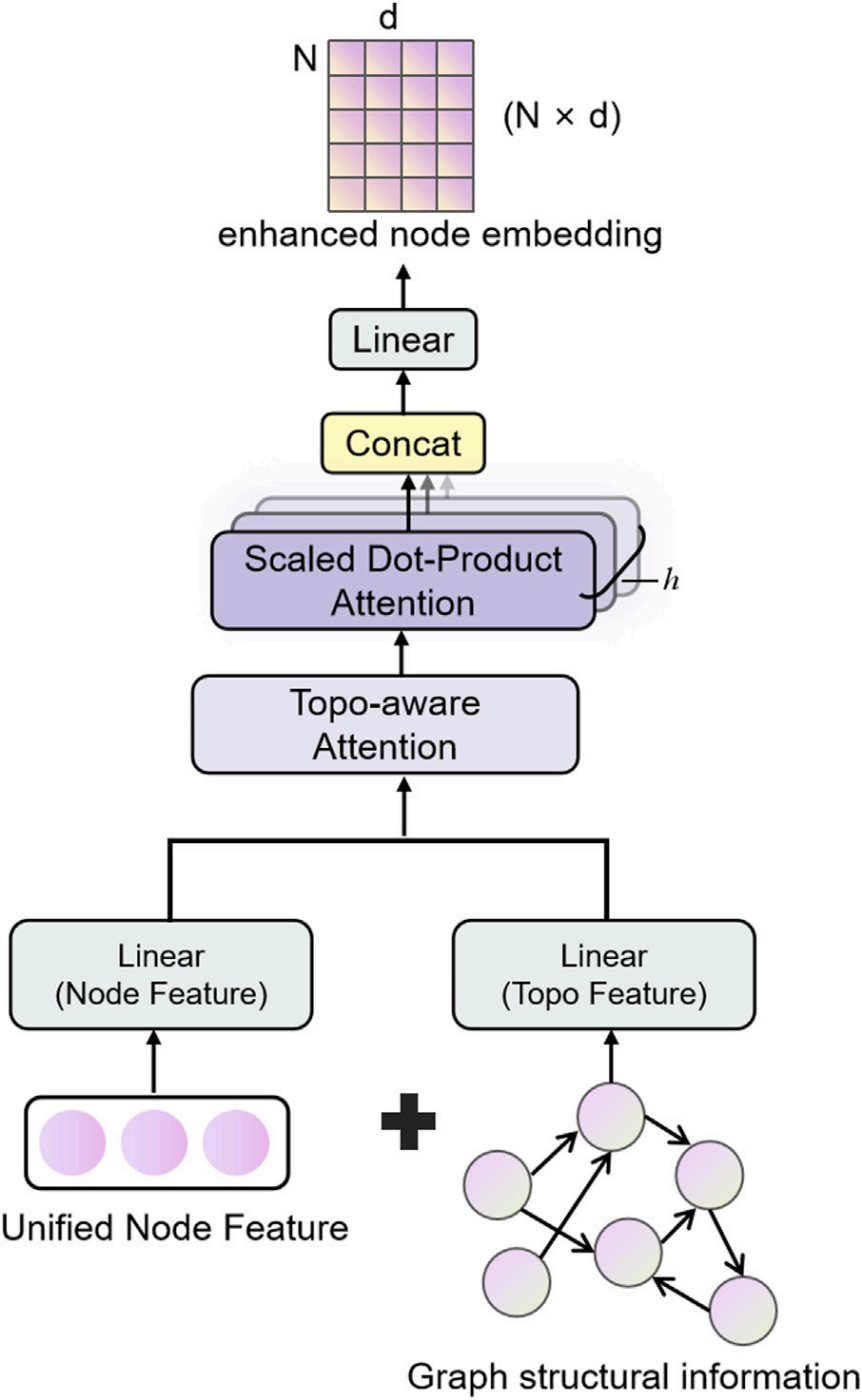
Schematic diagram of the structure of the Graph Topological Attention (GATA) module. Integrate node features and topological structure information, and enhance the graph structure modeling and feature expression capabilities through the topology-aware attention mechanism.

It is worth noting that the core mechanism of GTAT relies on the structural connections of the graph to assign attention weights, while the inclusion of topological features as auxiliary input is optional. In this study, we adopt a strategy that integrates both graph structure and topological information in the full model to maximize the representational capacity of the graph. The architecture of the proposed model is illustrated in Figure 2.

### 2.3 Feedforward network and residual connections

**FIGURE 3:**
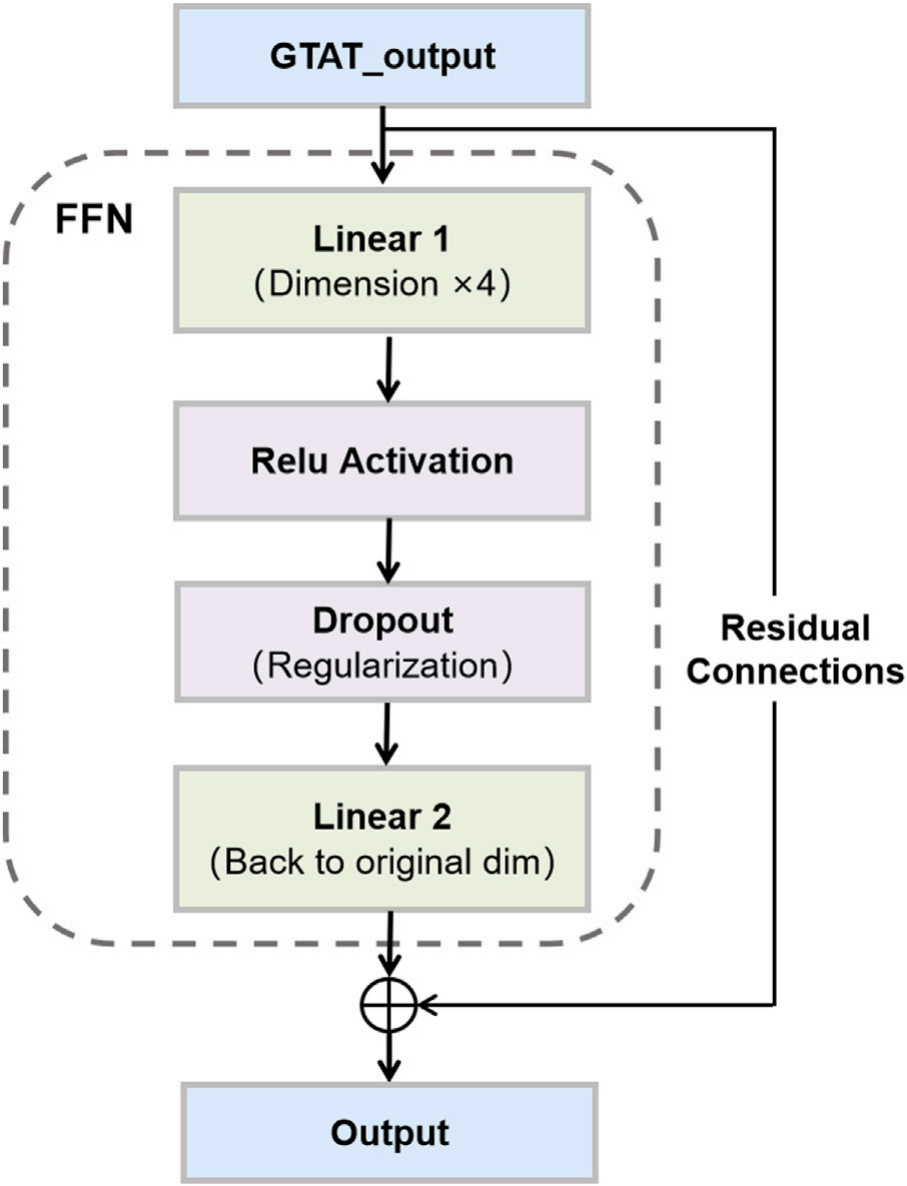
Feedforward Network and Residual Connections. This figure illustrates the structure of a feedforward network with skip connections, as used in our model.

Feedforward networks (FFN) and residual connections (RC) are common structural components in deep neural networks. In this model, FFN and residual connections together form a critical part of the neural network, facilitating effective gradient flow, mitigating the vanishing gradient problem, and accelerating convergence (Shefa et al., 2024), as shown in Figure 3. Particularly in GNNs, the design of FFNs and residual connections is crucial for handling complex graph-structured data.

#### 2.3.1 Feedforward network

In graph neural networks, feedforward networks (FFNs) are commonly employed to transform node features through non-linear mappings. In this study, we adopt a straightforward FFN architecture composed of two linear layers and a ReLU activation function. This structure enables the network to model complex, non-linear relationships among nodes, thereby enhancing representational capacity (Zhu et al., 2019). Specifically, each node’s input feature vector $h_{i}$ first undergoes a linear transformation, followed by a ReLU activation and another linear transformation to produce the updated feature representation. The computation process of the FFN is defined as:

$$h_{i}^{\prime }=W_{2}\cdot \text{ReLU}\left(W_{1}\cdot h_{i}+b_{1}\right)+b_{2}$$ (8)

Here, $h_{i}$ denotes the input features of node i , $W_{1}$ and $W_{2}$ are weight matrices, and $b_{1}$ , $b_{2}$ are bias terms. The ReLU activation function introduces non-linearity. The output $h_{i}^{\prime }$ represents the transformed node feature vector. Equation 8 captures the essential transformation pipeline of node-level feature encoding, allowing the network to better model higher-order dependencies in graph-structured data.

This structure enables the model to learn richer semantic representations from local node features, thereby enhancing its expressive power. Particularly when processing graph data, it can more flexibly adapt to varying node feature changes.

#### 2.3.2 Residual connections

In deep neural networks, increasing model depth often leads to issues such as vanishing or exploding gradients, which hinder effective training. To address this, residual connections (RCs) are widely adopted as a robust architectural enhancement (Guo and Sun, 2025). By introducing skip connections between layers, RCs allow input features to bypass nonlinear transformations and be directly added to the output, thereby improving gradient flow and facilitating model convergence. In our model, residual connections are applied after each feedforward network (FFN). Specifically, the input feature vector $h_{i}$ is added to its transformed version $h_{i}^{\prime }$ to obtain the final output. The computation process is defined as:

$$h_{i}^{\prime \prime }=h_{i}+h_{i}^{\prime }$$ (9)

Here, $h_{i}$ denotes the original node features, and $h_{i}^{\prime }$ is the final output feature. The final output $h_{i}^{\prime \prime }$ incorporates both the transformed and original information.

As shown in Equation 9, residual connections help preserve the original feature context while enabling deeper representations, thereby enhancing model stability and training efficiency (Wang et al., 2024).

#### 2.3.3 Synergy between feedforward networks and residual connections

The combination of feedforward networks and residual connections significantly enhances the network’s expressive power and training efficiency. Feedforward networks transform node features through two fully connected layers, enhancing feature representation ability. However, without residual connections, deep networks may cause excessive information transformation, leading to instability during training. Introducing residual connections ensures the continuity of information at each layer, helping stabilize gradient flow, prevent vanishing gradients, and accelerate convergence during training. The specific advantages are as follows.

1. Mitigating the Vanishing Gradient Problem: As the network depth increases, gradients in deep neural networks may gradually vanish, impacting training performance. Residual connections, through skip connections, allow information to propagate across multiple layers, maintaining gradient flow and mitigating the vanishing gradient issue, thus enhancing network stability (Zhou et al., 2024).
2. Accelerating Convergence: Residual connections enable direct input-output relationships at each layer, speeding up gradient updates and improving convergence speed (Nie et al., 2019), especially in deep networks.
3. Enhancing Model Expressiveness: Feedforward networks enhance node feature representation through non-linear transformations, enabling the network to capture more complex feature relationships. Residual connections ensure effective information flow, preventing inter-layer information loss, further enhancing the model’s learning capacity (Belhaouari and Kraidia, 2025).
4. Improving Robustness: The combination of feedforward networks and residual connections enhances the model’s adaptability to noise and complex graph structures. Residual connections ensure that information is not excessively distorted after multiple layer stacks, enhancing the model’s robustness and noise resistance (Pan et al., 2025).

### 2.4 Output layer

**FIGURE 4:**
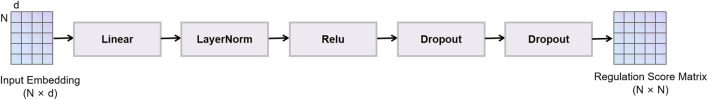
Output layer.

In this model, the output layer (Figure 4) maps node representations processed by graph convolution, feature fusion, and the feedforward network to regulatory probability scores between genes. This layer first reduces the dimensionality of fused node features via a linear transformation, then applies layer normalization, ReLU activation and Dropout regularization in sequence to enhance the model’s expressiveness and generalization (Reddy et al., 2024). During training we use Focal Loss as the objective function to mitigate class imbalance and improve the model’s ability to detect rare regulatory interactions. The raw output is a real-valued score, where values above zero indicate a predicted regulatory interaction and values at or below zero indicate no interaction. The magnitude of this score reflects the model’s confidence. During inference the raw score is passed through a Sigmoid function to map it into the [0, 1] interval and yield a probability for regulatory interaction.

### 2.5 Dataset

To evaluate the effectiveness of the proposed GTAT-GRN method for GRN inference, we employed both simulated benchmark datasets and real biological expression data. The simulated dataset is DREAM4 InSilico_Size100 (Marbach et al., 2010), generated by the GeneNetWeaver (GNW) tool (Schaffter et al., 2011), which accurately simulates transcriptional regulatory mechanisms and provides a known gold standard network. This dataset is widely recognized as a standard benchmark in GRN inference research (Marbach et al., 2012). It contains five independent sub-networks of 100 genes each, covering diverse experimental conditions such as time-series perturbations and homeostasis interventions (Marbach et al., 2009). The time-series data include 21 equally spaced sampling points with intervals of 50 time units. For feature extraction, we used a sliding window with size 3 and step size 1. The real dataset is DREAM5 *Escherichia coli* (*Escherichia coli*) expression data (Lim et al., 2013), derived from experiments that include time-series measurements and gene knockouts. It also provides an official gold standard network, enabling performance evaluation under real biological conditions. This dataset contains 4,511 genes, of which 1,371 exhibit time-series characteristics. We selected these genes for experiments and applied a sliding window with size 5 and step size 1 during feature extraction.

Through systematic experiments on these datasets, we comprehensively evaluate the ability of GTAT-GRN to uncover potential regulatory relationships under limited-sample conditions, and verify its adaptability and robustness in reconstructing complex network structures. Detailed dataset information is provided in Table 2.

**TABLE 2:** Dataset.

Dataset	Network	Gene	Expression data	Known regulatory interaction
DREAM4	Net 1	100	210	176
	Net 2	100	210	249
	Net 3	100	210	195
	Net 4	100	210	211
	Net 5	100	210	211
DREAM5	E.coli	4,511	805	2066

## 3 Results

### 3.1 Performance metrics

To evaluate the performance of the proposed method in GRN reconstruction, we adopt two widely used metrics as the primary evaluation indicators (Aalto et al., 2020): the area under the receiver operating characteristic curve (AUC) and the area under the precision–recall curve (AUPR). AUC is computed as the area under the ROC curve, which depicts the trade-off between the true positive rate (TPR) and the false positive rate (FPR). AUPR measures the area under the precision–recall (PR) curve, which illustrates the balance between precision and recall.

The relevant metrics are defined as follows:

$$\text{TPR}=\frac{\text{TP}}{\text{TP}+\text{FN}}$$ (10)

$$\text{FPR}=\frac{\text{FP}}{\text{FP}+\text{TN}}$$ (11)

$$\text{Precision}=\frac{\text{TP}}{\text{TP}+\text{FP}}$$ (12)

$$\text{Recall}=\frac{\text{TP}}{\text{TP}+\text{FN}}$$ (13)

$$F1=2\times \frac{\text{Precision}\times \text{Recall}}{\text{Precision}+\text{Recall}}$$ (14)

In Equations 10–14, TP (true positives) denotes the number of correctly identified regulatory links, TN (true negatives) denotes correctly identified non-links, FP (false positives) represents incorrectly predicted links, and FN (false negatives) corresponds to missed regulatory interactions. These metrics collectively reflect the model’s capability in both identifying true regulatory edges and avoiding false predictions.

### 3.2 Experimental results

To evaluate the overall performance of the proposed GTAT-GRN model in gene regulatory network (GRN) inference, we conducted comprehensive training and testing procedures on five independent subnetworks provided by the DREAM4 InSilico Size100 dataset. The performance of the model was measured by multiple indicators such as AUC, AUPR, Precision@k, Recall@k and F1-score@k.

**FIGURE 5:**
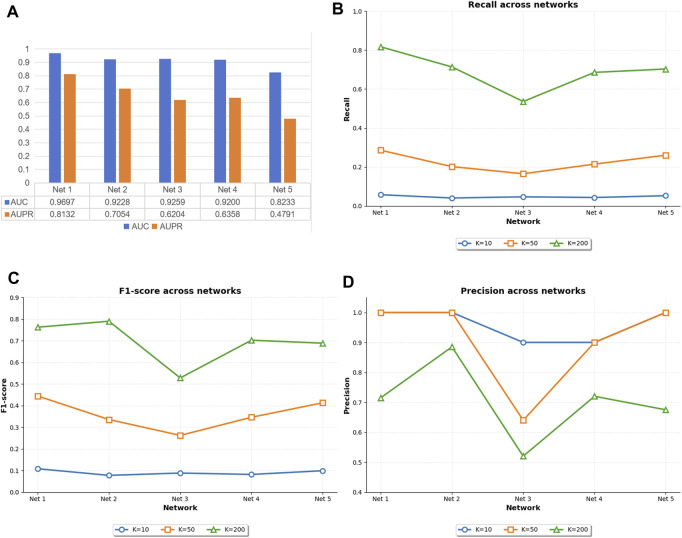
The performance of GTAT-GRN on five datasets of DREAM4. **(A)** AUC and AUPR performance of GTAT-GRN on DREAM4 **(B)** Recall@k of GTAT-GRN on DREAM4 **(C)** F1@k of GTAT-GRN on DREAM4 **(D)** GTAT-GRN at Precision@k in DREAM4.

Figure 5 illustrates the original experimental results of GTAT-GRN on the five subnetworks of DREAM4. As shown, GTAT-GRN consistently achieved stable and outstanding performance on all datasets, demonstrating its adaptability and robustness under diverse regulatory scenarios. The model performed particularly well on E. coli Network 1, achieving an AUC of 0.9697 and an AUPR of 0.8132, indicating its strong capability in capturing complex regulatory structures. In contrast, performance on *S. cerevisiae* Network 5 was relatively weaker. This discrepancy can be attributed to notable differences in the topological structures and sample distributions of the two networks. Specifically, E. coli Network 1 contains more edges and exhibits higher structural density, which facilitates effective information propagation and feature integration through graph neural networks. Moreover, its relatively balanced label distribution enables the model to learn distinctions between different classes more effectively during training, thereby enhancing generalization. On the other hand, *S. cerevisiae* Network 5 has fewer edges and more sparse structural information, limiting the propagation of topological features. Its severely imbalanced label distribution also increases the risk of overfitting and weakens the model’s ability to infer regulatory relationships, ultimately compromising AUC and AUPR performance.

**FIGURE 6:**
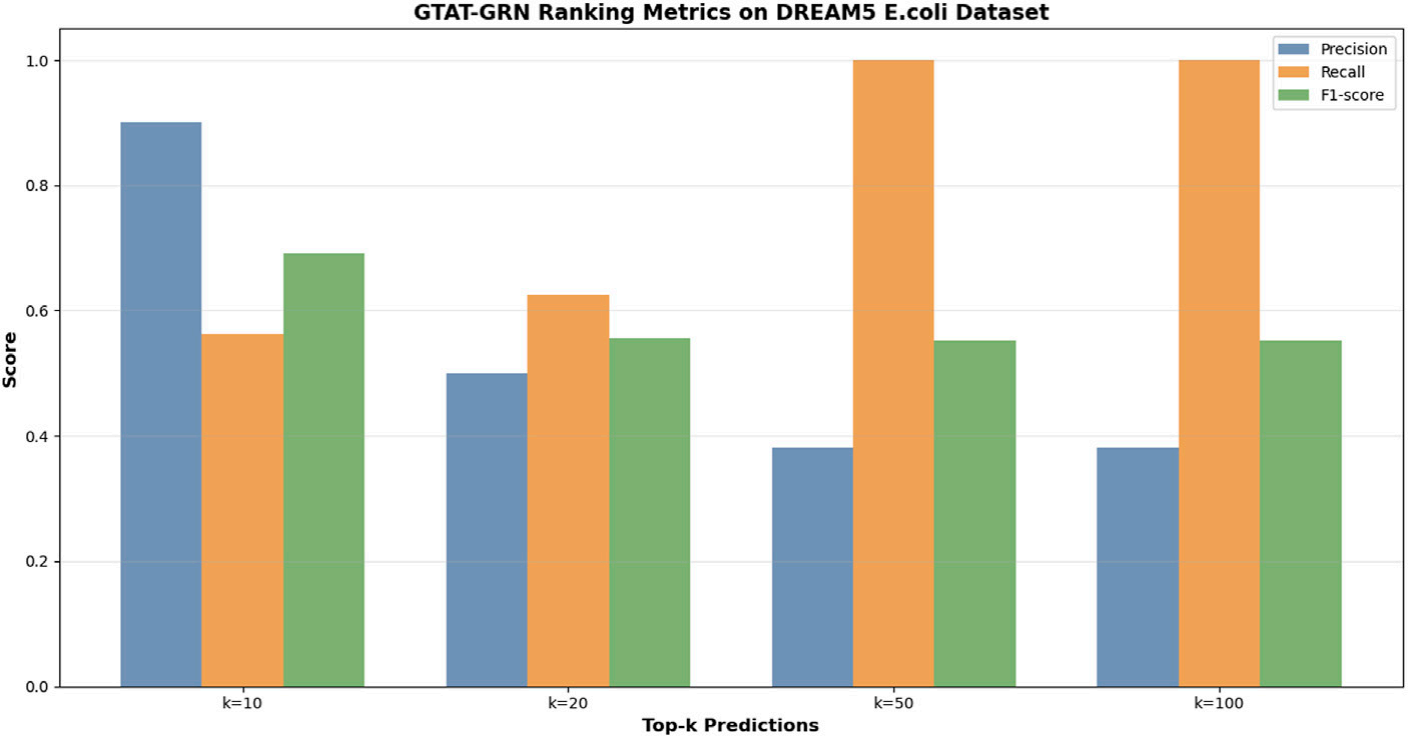
The performance of GTAT-GRN on E. coli datasets of DREAM5.

Figure 6 presents the experimental results of GTAT-GRN on the DREAM5 *E. coli* dataset, demonstrating its strong performance in gene regulatory network inference. The model achieved an AUC of 0.7596 and an AUPR of 0.6075, indicating robust overall performance. Notably, for high-confidence predictions, Precision@10 reached 0.9000 and F1@10 was 0.6923, suggesting that the model can accurately identify the most reliable regulatory relationships. Meanwhile, the Recall value demonstrates the model’s excellent coverage ability. These results verified the validity and practicability of GTAT-GRN on real biological data. These results confirm the validity and practical applicability of GTAT-GRN on real biological data.

In summary, the experimental results further confirm the critical roles of graph structural density and label balance in GRN inference, indicating the performance advantages and practical application potential of GTAT-GRN in modeling structural dependencies and adapting to diverse data distributions.

### 3.3 Model performance comparison

To comprehensively evaluate the performance of the proposed model in gene regulatory network inference, we selected eight representative baseline methods on DREAM4 InSilico_Size100: traditional statistical approaches (e.g., PCA-CMI (Zhao et al., 2016)), information-theoretic algorithms (GENIE3 and dynGENIE3 (Huynh-Thu and Geurts, 2018)), dynamic modeling techniques (NSRGRN (Liu et al., 2023)), and recent graph neural network–based methods (NIMEFI (Ruyssinck et al., 2014), MMFGRN (He et al., 2021), and GreyNet (Chen and Liu, 2022), DGCGRN (Wei et al., 2024)). We conducted a systematic comparative analysis on DREAM4’s five benchmark networks (Net1–Net5), using AUC, AUPR, Precision@k, Recall@k and F1-score@k as evaluation metrics to assess and contrast each method’s ability to reconstruct regulatory interactions.

**FIGURE 7:**
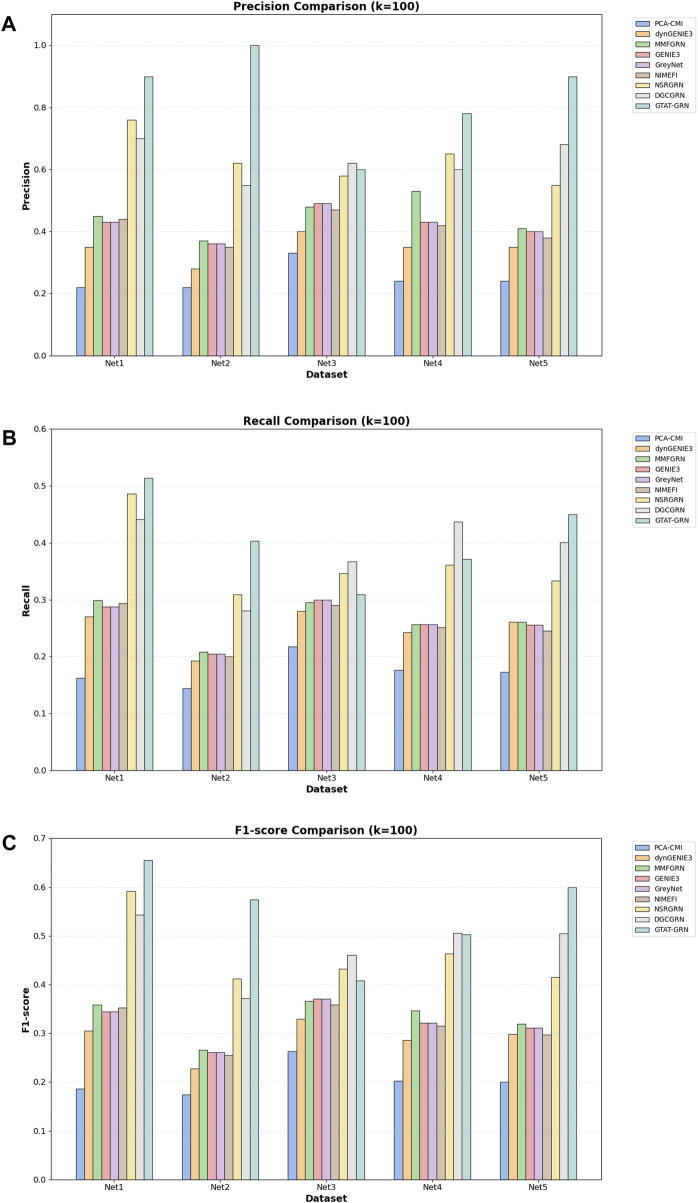
Comparison of different methods on the DREAM4 dataset **(A)** comparison of models on Precision@100 **(B)** comparison of models on Recall@100 **(C)** comparison of models on F1-score@100.

As shown in Table 3, the proposed GTAT-GRN model achieved overall superior AUC and AUPR performance across the five standard datasets, fully demonstrating its comprehensive performance advantages in the task of gene regulatory relationship identification. Experimental results indicate that the introduction of the graph topology attention mechanism, combined with the multi-source feature fusion strategy, enables the model to more comprehensively characterize the complex regulatory relationships between genes, thereby significantly improving the accuracy and stability of network structure inference. Particularly, compared with traditional methods and existing GNN models, GTAT-GRN exhibits stronger representation capability in modeling long-range regulatory paths and capturing dynamic expression features. To provide a more detailed assessment of high-confidence predictions, we evaluated Precision@k, Recall@k, and F1-score@k for K = 10, 50, and 100. In the main text, we present the results for K = 100 as a representative value, which balances high-confidence prediction and overall coverage. Results for other K values are provided in the Supplementary Figures S1–S6. The results for K = 100 in Figure 7 indicate that GATA-GRN achieves superior coverage and accuracy, further validating its robustness and potential for real-world applications.

**TABLE 3:** Performance comparison (AUC and AUPR) of various methods on five networks (Net1–Net5).

Method	Net1		Net2		Net3		Net4		Net5	
	AUC	AUPR	AUC	AUPR	AUC	AUPR	AUC	AUPR	AUC	AUPR
dynGENIE3	0.7851	0.1888	0.6851	0.1019	0.7459	0.1685	0.7311	0.1685	0.7728	0.1774
PCA-CMI	0.7902	0.0700	0.6955	0.0994	0.7808	0.1287	0.7493	0.0916	0.7028	0.0623
MMFGRN	0.8014	0.3375	0.7329	0.2315	0.7611	0.3397	0.7643	0.3166	0.7623	0.2383
GENIE3	0.8216	0.0964	0.7651	0.1041	0.8240	0.1414	0.8299	0.1471	0.7901	0.1510
GreyNet	0.8222	0.2580	0.7251	0.1603	0.7712	0.2674	0.7316	0.2057	0.7830	0.2210
NIMEFI	0.8470	0.1071	0.7942	0.1254	0.8404	0.1731	0.8246	0.1463	0.7671	0.0977
NSRGRN	0.9043	0.5366	0.7787	0.3324	0.8150	0.3671	0.8324	0.3996	0.7504	0.1754
DGCGRN	0.8820	—	0.7965	—	0.8911	—	0.8667	—	**0.8905**	—
GATA-GRN	**0.9697**	**0.8132**	**0.9228**	**0.7054**	**0.9259**	**0.6204**	**0.9200**	**0.6358**	0.8233	**0.4791**

Bold indicates the best results, and underlined values represent the second-best results.

However, on the Net5 dataset (i.e., *S. cerevisiae* Network 5), the AUC value of DGCGRN exceeded that of the proposed method. This result may be attributed to the significant class imbalance in the dataset, where certain regulatory relationship samples are relatively scarce, leading the model to be biased towards the majority class during training. In such scenarios, the conditional variational autoencoder (CVAE) enhancement mechanism introduced in DGCGRN Wei et al. (2024) can effectively model the potential distribution of adjacent nodes conditioned on the central node features, thereby enhancing the representation capability for low-degree nodes. Additionally, DGCGRN integrates sequential features extracted via Bi-GRU and statistical features, strengthening its modeling capacity for regulatory dependency structures, which contributes to its superior performance on Net5.

Nevertheless, on the remaining four datasets, GTAT-GRN still demonstrates stronger adaptability and robustness. Benefiting from the synergistic effect of the graph topology attention mechanism and multi-source information fusion strategy, the proposed method can effectively capture complex regulatory structures and latent expression patterns without relying on specific enhancement mechanisms, showing broader applicability and higher stability.

To further assess the applicability of GATA-GRN in real biological gene regulatory scenarios, we performed comparative experiments on the DREAM5 *E. coli* gene expression dataset, which originates from actual *E. coli* experiments. We selected several representative baseline methods, including dynGENIE3 Huynh-Thu and Geurts (2018) (a random forest-based temporal regulation inference method), GreyNet Chen and Liu (2022) (a graph neural network-based inference framework), and MMFGRN Wei et al. (2024) (a multimodal feature fusion method). These methods represent three typical strategies: traditional machine learning, graph deep learning, and multimodal fusion.

**FIGURE 8:**
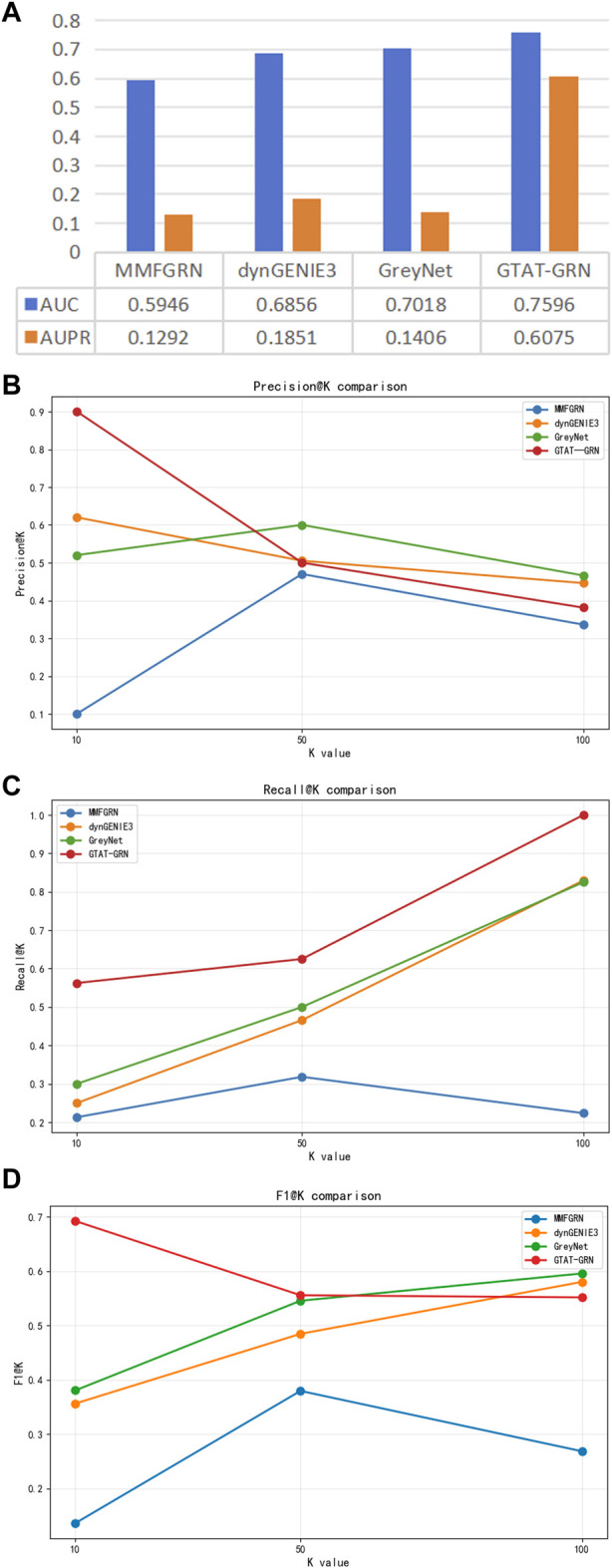
Comparison of different methods on the DREAM5 E.coli dataset. **(A)** Comparison of AUC and AUPR of the model in E.coli **(B)** comparison of Precision@k of the model in E.coli **(C)** comparison of Recall@k of the model in E.coli **(D)** Comparison of the model in E.coli at F1-score@k.

As shown in Figure 8, GATA-GRN outperformed existing methods in AUC and AUPR on the *E. coli* dataset, particularly excelling at capturing dynamic gene dependencies and long-range regulatory interactions. Moreover, GATA-GRN exhibited notable improvements in key metrics, including Precision@k, Recall@k, and F1-score@k, further validating the robustness and practical potential of the proposed method in real-world scenarios.

### 3.4 Ablation experiment

In our ablation experiments, we use the E. coli1 dataset and adopt a simplified model consisting solely of the Graph Convolutional Network (GCN) backbone as the baseline. To systematically assess the individual and combined contributions of each component to inference performance, we incrementally introduce the FFN and Residual, GTAT, and Topo modules, recording the AUC, AUPR, Precision@k, Recall@k and F1-score@k performance of the model at each step. As shown in Table 4, each architectural component contributes positively to model performance. Starting from the simplified GCN backbone, the introduction of the FFN and residual connections significantly enhances feature representation and training stability, yielding steady improvements in both AUC and AP. Adding the GTAT module further boosts performance by effectively modeling directional regulation and long-range dependencies. Notably, GTAT—relying solely on the graph’s structural connectivity edge_index for attention weighting—remains highly effective even without explicit topological features. Finally, incorporating explicit topological features as structural priors achieves the highest performance, demonstrating that the topology-aware attention mechanism and topological information complement each other in capturing gene regulatory dependencies.

**TABLE 4:** Ablation study results on the E.coli1 dataset.

	AUC	AP	F1@100	Recall@100	Precision@100
Exp 1. Base	0.6352	0.5759	0.3940	0.3171	0.5200
Exp 2. +ResFFN	0.7679	0.6535	0.4218	0.3314	0.5800
Exp 3. +ResFFN + GTAT	0.8335	0.6671	0.6737	0.5314	0.9200
Exp 4. +ResFFN + GTAT + Topo	0.9697	0.8132	0.6545	0.5143	0.9000

### 3.5 Cross-validation experiment

To further evaluate the validity and robustness of the GTAT-GRN model under varying network structures and sample distributions, we conducted five-fold cross-validation experiments on three representative subsets from the DREAM4 InSilico dataset: E. coli Network 1, E. coli Network 2, and *S. cerevisiae* Network 5. Given the significant differences among these datasets in terms of class imbalance, edge density, and graph complexity, we did not adopt a unified data split ratio. Instead, we tailored the proportions of training, validation, and testing sets for each subset based on their specific characteristics. For instance, in E. coli Network 1, which contains relatively fewer positive samples, we increased the proportion of training data to enhance the model’s ability to learn from rare examples. In contrast, for E. coli Network 2 and *S. cerevisiae* Network 5, which exhibit more pronounced class imbalance, a more balanced partitioning strategy was employed to ensure representative coverage of each class during model training and to guarantee fair and valid evaluation.

In addition, to ensure consistency in sample distribution across folds, we applied stratified sampling during the splitting process and fixed the random seed (set to 42) to enhance reproducibility. Specifically, positive and negative samples were split and shuffled separately before being combined into fold-specific datasets, thereby ensuring fairness and representativeness in the cross-validation procedure.

The cross-validation results (Tables 5–7) show that GTAT-GRN consistently achieves high performance across multiple folds, demonstrating both stability and structural adaptability. Specifically, E. coli1 achieved an average AUC of 0.9344 ± 0.0148 and an AUPR of 0.5976 ± 0.0811. For E. coli2, the average AUC was 0.9637 ± 0.0024, with an AUPR of 0.7682 ± 0.0181.*S. cerevisiae*5 achieved an AUC of 0.8631 ± 0.0172 and an AUPR of 0.3422 ± 0.0395. In addition, the model showed stable performance in Top-100 Precision, Recall, and F1 scores. Compared with the fixed-split results of the original experiment, the cross-validation results for E. coli1 and E. coli2 showed only minor differences, confirming the consistency and stability of model performance. Results for *S. cerevisiae*5 fluctuated slightly but remained at a relatively high level overall.

**TABLE 5:** E.coli1 cross-validation results.

Fold	AUC	AUPR	Precision@100	Recall@100	F1@100
Fold 1	0.9232	0.5727	0.8500	0.4830	0.6163
Fold 2	0.9464	0.6537	0.9200	0.5227	0.6667
Fold 3	0.9479	0.6897	0.9300	0.5284	0.6738
Fold 4	0.9107	0.4553	0.8700	0.4943	0.6309
Fold 5	0.9437	0.6166	0.9100	0.5170	0.6596

**TABLE 6:** E.coli2 cross-validation results.

Fold	AUC	AUPR	Precision@100	Recall@100	F1@100
Fold 1	0.9655	0.7865	0.9000	0.3936	0.5476
Fold 2	0.9604	0.7380	0.9200	0.4016	0.5592
Fold 3	0.9668	0.7838	0.9200	0.4016	0.5592
Fold 4	0.9614	0.7746	0.9000	0.4016	0.5554
Fold 5	0.9643	0.7581	0.9300	0.4016	0.5610

**TABLE 7:** S.cerevisiae5 Cross-Validation results.

Fold	AUC	AUPR	Precision@100	Recall@100	F1@100
Fold 1	0.8685	0.3475	0.9000	0.4830	0.6279
Fold 2	0.8368	0.2746	0.9200	0.5227	0.6663
Fold 3	0.8770	0.3896	0.9600	0.5284	0.6811
Fold 4	0.8832	0.3701	0.9400	0.4943	0.6482
Fold 5	0.8501	0.3293	0.9500	0.5170	0.6689

Overall, these cross-validation findings confirm GTAT-GRN’s robustness and ability to generalize under varying sample distributions, laying a solid foundation for its application to diverse biological network modeling tasks.

## 4 Discussion and conclusion

This study presents GTAT-GRN, a deep graph neural network that incorporates a graph topological attention mechanism and integrates multi-source features. Experimental results indicate that GTAT-GRN effectively captures complex regulatory dependencies and exhibits robust and stable performance across benchmark datasets, including DREAM4 and DREAM5. Compared to traditional graph neural networks (e.g., GCN and GAT), GTAT-GRN’s main advantage is its explicit modeling of topological structures. Classical GAT primarily considers symmetrical attention distribution among node features, whereas regulatory relationships in GRNs are inherently directional and asymmetric (Ali et al., 2020). The GTAT module, with its unique structural design, better captures this biological property, which may explain its improved performance.

Comparison with contemporary advanced methods (e.g., DGCGRN) highlights the interaction between model properties and data structure. For example, on the Nte5 dataset of DREAM4, DGCGRN slightly outperforms GTAT-GRN, likely due to the CVAE mechanism’s advantage in processing low-connectivity nodes (Wei et al., 2024). Nevertheless, across most datasets, GTAT-GRN shows superior and more stable performance, suggesting that its multi-source feature fusion strategy enhances generalization and is less affected by dataset-specific structural biases.

Compared with tree-based and information-theory methods (e.g., GENIE3, dynGENIE3 (Huynh-Thu and Geurts, 2018), PCA-CMI (Zhao et al., 2016)), GTAT-GRN excels in representation learning. While GENIE3 and its variants can capture nonlinear relationships effectively, they rely on feature importance rankings and cannot explicitly model topological gene dependencies. PCA-CMI may suffer from insufficient statistical power when analyzing high-dimensional data. In contrast, GTAT-GRN generates context-rich node representations and directly infers regulatory relationships. GreyNet also infers GRNs from time-series expression data, but its performance is constrained by underlying assumptions.

Despite demonstrating effectiveness in reconstructing complex gene regulatory structures on benchmark datasets, GTAT-GRN has several areas for further improvement. First, the model does not yet fully differentiate regulatory relationship types, such as activation and inhibition. Future work could incorporate symbolic information or positive/negative regulatory labels. Second, the model still depends on predefined topological features. Future studies may develop structurally adaptive GNN mechanisms to enable automatic feature learning and reduce reliance on manual feature engineering.

Moreover, applying the model to single-cell RNA-seq (scRNA-seq) or spatial transcriptomics (ST) data requires addressing inherent challenges: scRNA-seq data exhibit high sparsity, dropout events, significant cellular heterogeneity, and batch effects (Wang et al., 2023); ST data additionally show spatial autocorrelation and resolution limitations (Zhao et al., 2024). GTAT-GRN’s core advantage is its graph topological attention mechanism with multi-source feature integration, offering a novel approach for analyzing such data. For example, single cells or spatial locations can be represented as graph nodes to capture complex interactions. This framework inherently supports joint modeling of gene expression, cell type annotation, spatial coordinates, and multi-omics data, enabling comprehensive characterization of complex regulatory patterns.

Compared with methods for inferring gene co-expression or co-relationships (e.g., COTAN (Galfrè et al., 2021), scGeneClust (Deng et al., 2023), CS-Core (Su et al., 2023), LEGEND (Deng et al., 2025)), GTAT-GRN theoretically benefits from jointly leveraging topological structures and multi-source contextual features. This approach is more capable of uncovering complex regulatory pathways across cell types or spatial domains. In particular, in ST scenarios, studies such as STANDS (Xu et al., 2024) have demonstrated spatial expression heterogeneity of tumor-associated genes across anatomical regions. Future extensions of GTAT-GRN could compare GRN structural differences between normal and tumor regions within the same tissue section, offering novel computational perspectives and biological insights into tumor microenvironment regulatory disruptions.

In summary, GTAT-GRN offers a novel, structurally-informed framework for inferring gene regulatory networks. Experimental results confirm its strong performance and broad applicability in inferring complex regulatory structures. Future studies could explore multi-omics data integration, real-world validation, and enhanced model interpretability to further advance GTAT-GRN’s application in precision medicine and systems biology.

## Data Availability

The original contributions presented in the study are included in the article/Supplementary Material, further inquiries can be directed to the corresponding author.

## Supplementary material

Supplementary Figure S1 Recall@50 of GTAT-GRN on DREAM4.

Supplementary Figure S2 Precision@50 of GTAT-GRN on DREAM4.

Supplementary Figure S3 F1@50 of GTAT-GRN on DREAM4.

Supplementary Figure S4 Recall@10 of GTAT-GRN on DREAM4.

Supplementary Figure S5 Precision@10 of GTAT-GRN on DREAM4.

Supplementary Figure S6 F1@10 of GTAT-GRN on DREAM4.
