# Supplementary figures and images for: GTAT-GRN: a graph topology-aware attention method with multi-source feature fusion for gene regulatory network inference
Source: Front Genet. 2025 Oct 8;16:1668773. doi: 10.3389/fgene.2025.1668773 (PMC12540167; doi:10.3389/fgene.2025.1668773)

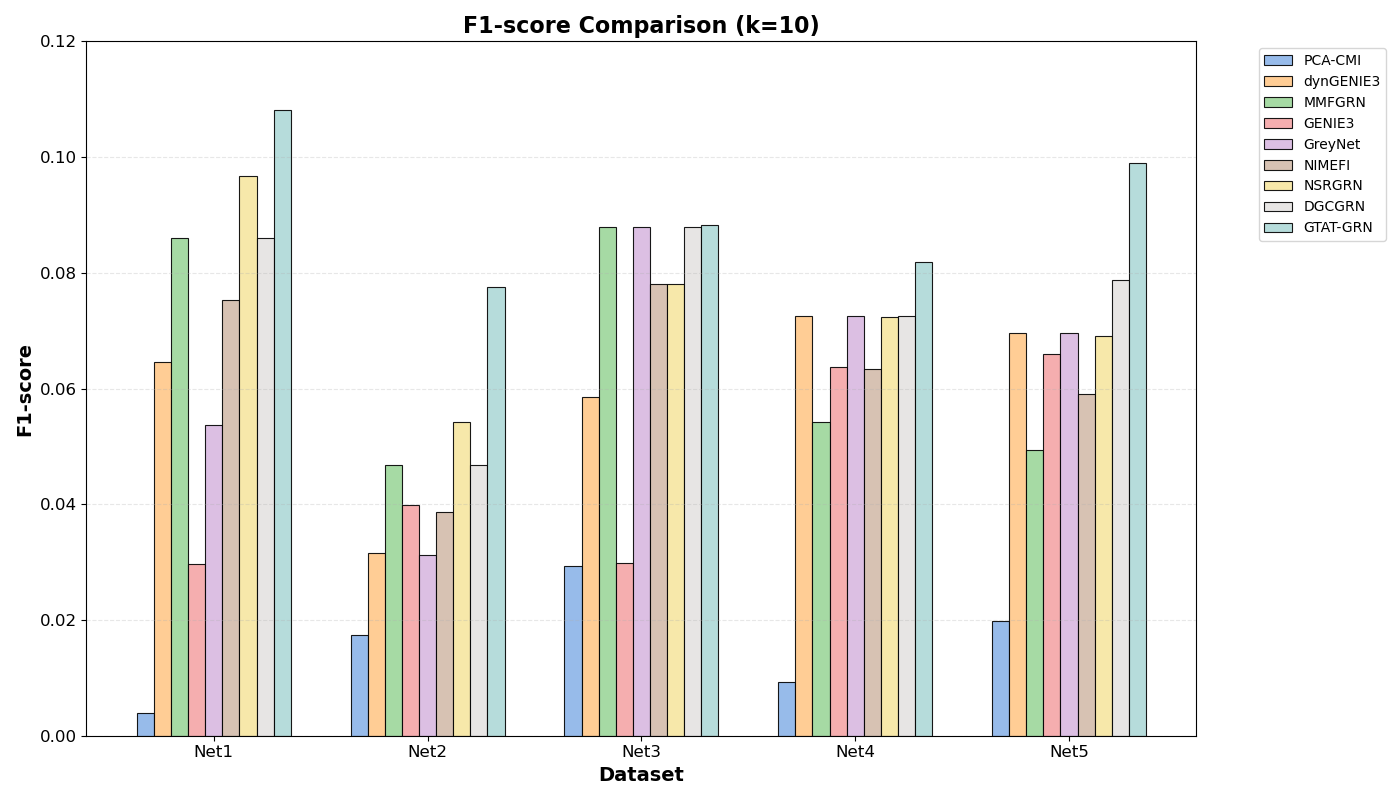

Supplement: Supplementary file 1 [file Image6.tif]

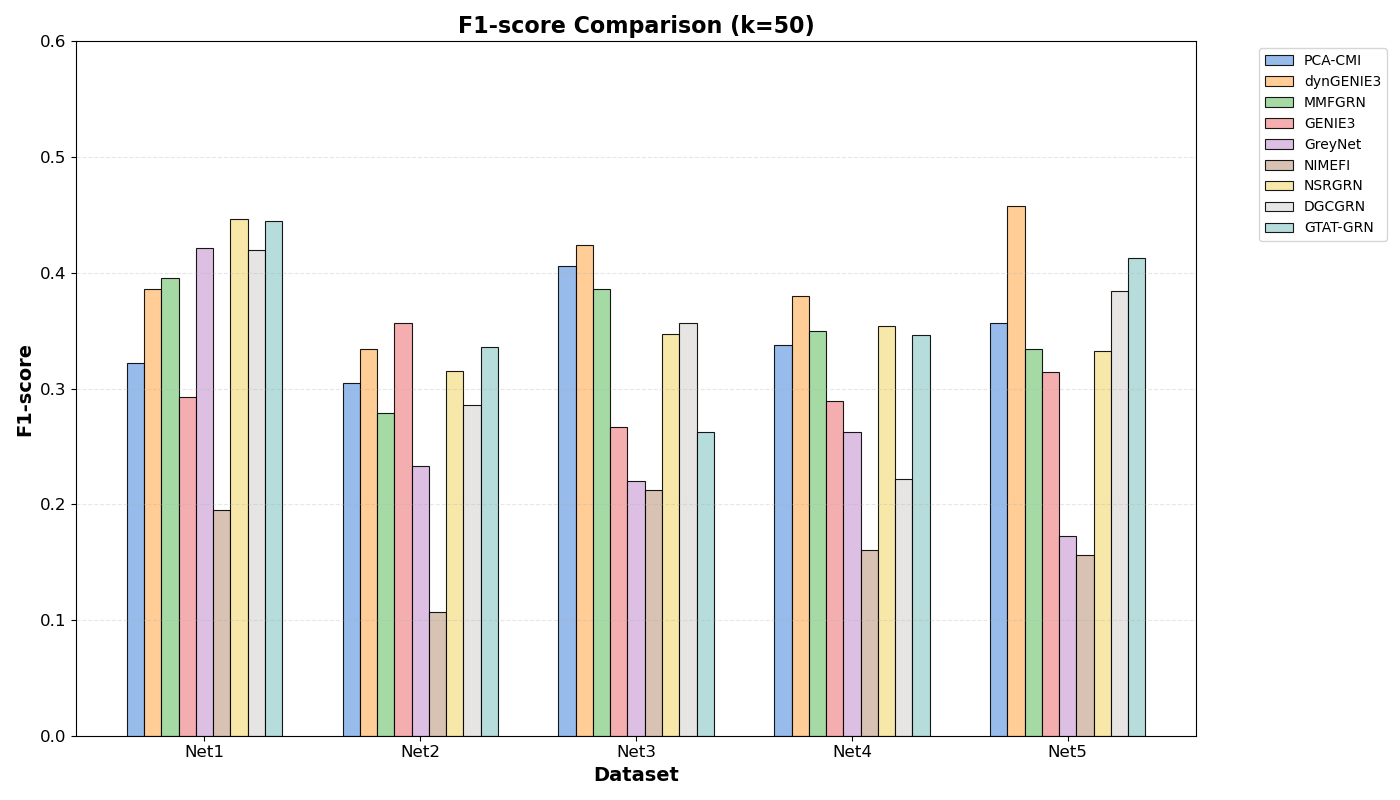

Supplement: Supplementary file 2 [file Image3.tif]

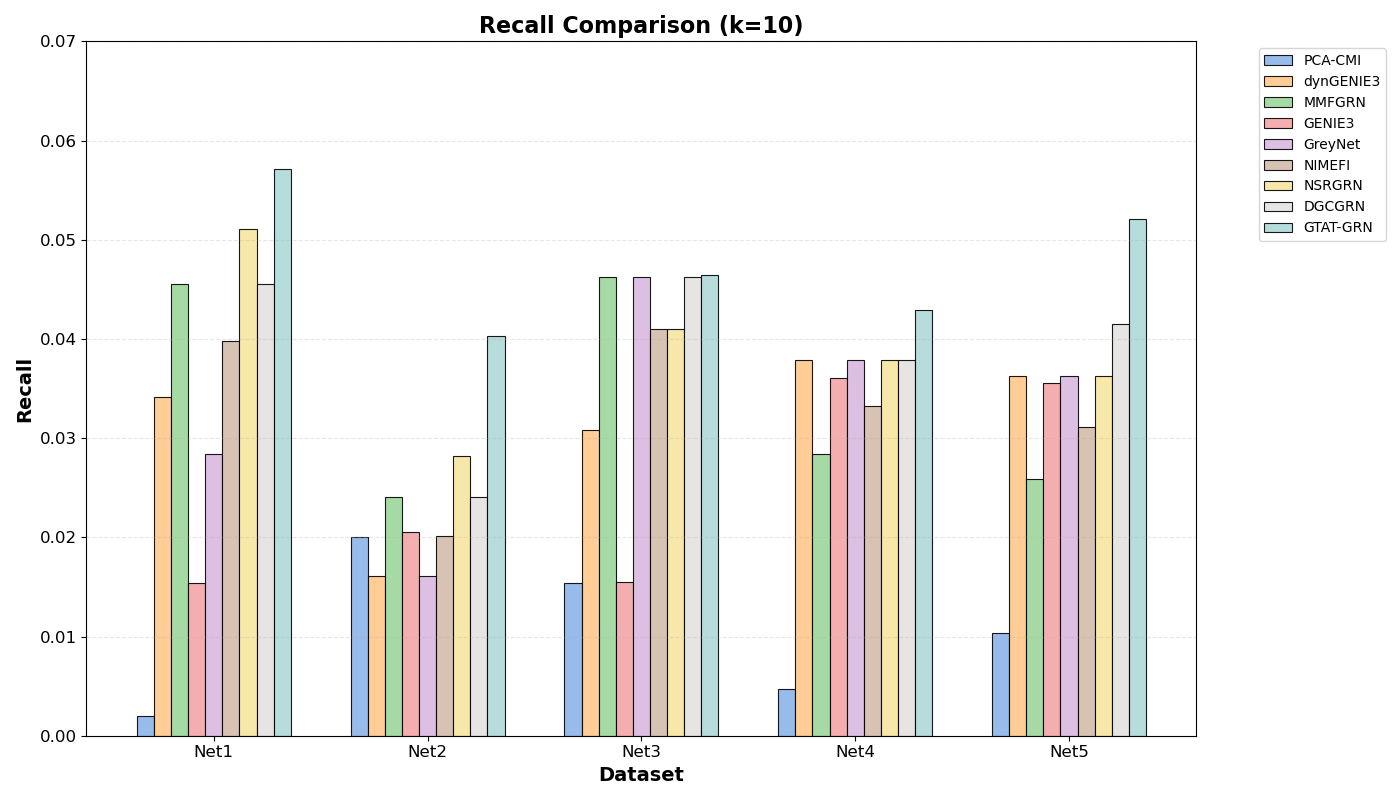

Supplement: Supplementary file 3 [file Image4.tif]

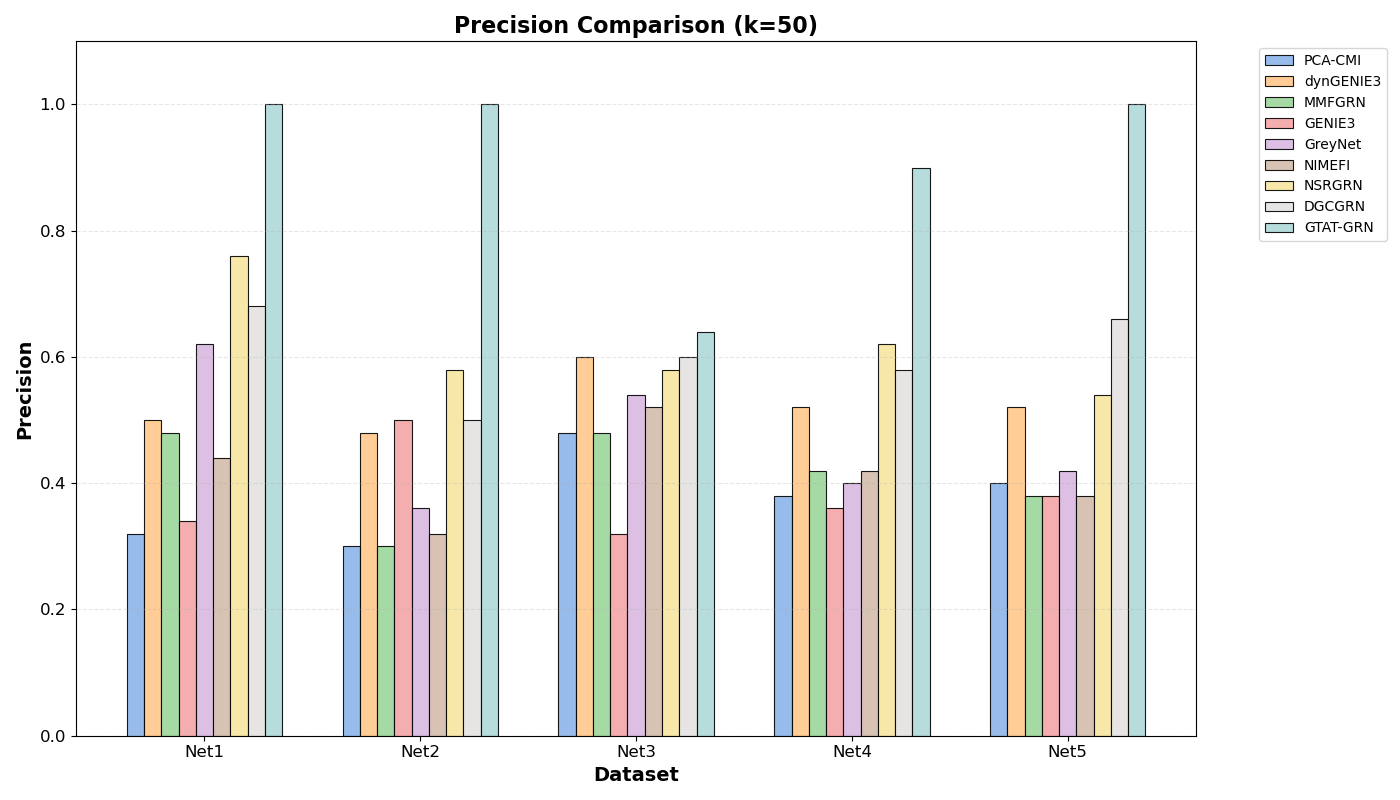

Supplement: Supplementary file 4 [file Image2.tif]

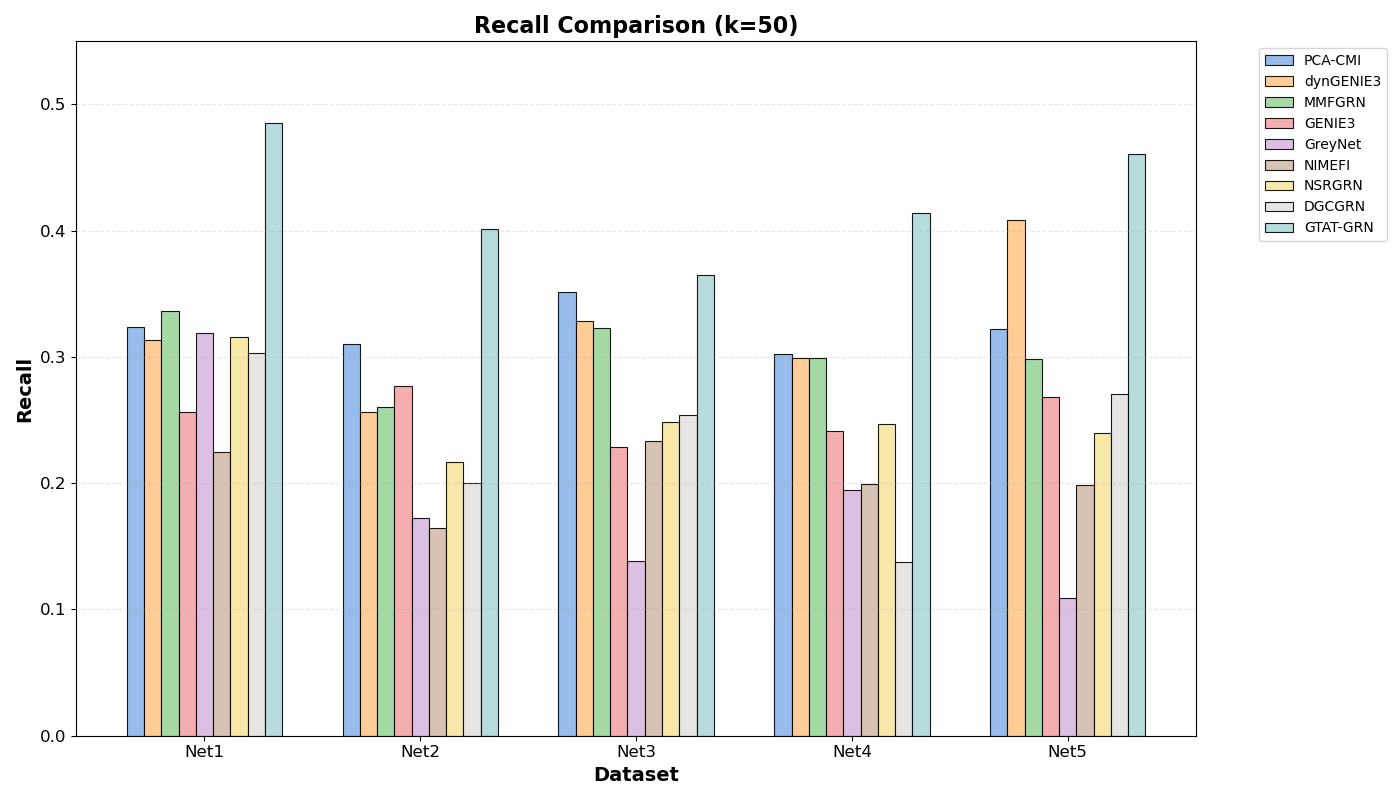

Supplement: Supplementary file 5 [file Image1.tif]

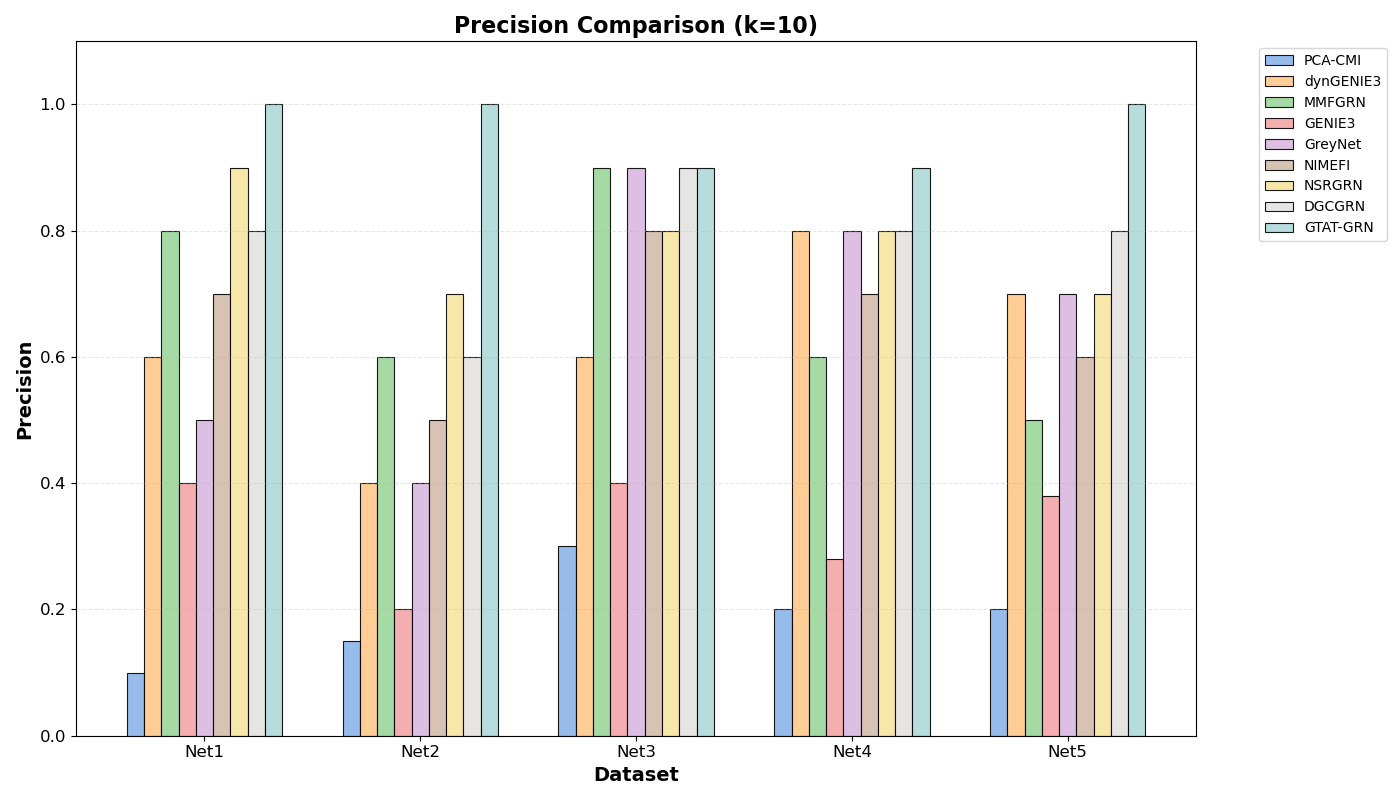

Supplement: Supplementary file 6 [file Image5.tif]
